# Enhanced rotator cuff tendon-bone interface regeneration with injectable manganese-based mesoporous silica nanoparticle-loaded dual crosslinked hydrogels

**DOI:** 10.3389/fbioe.2025.1645970

**Published:** 2025-08-21

**Authors:** Zihang Chen, Youjie Liu, Tianxiang Liang, Zhaoyuan Du, Liming Deng, Zhiwen Wu, Ye Li, Haobo Zhong, JinJin Ma, Riwang Li, Huajun Wang, Qiu Dong, Tao Yu, Xiaofei Zheng

**Affiliations:** ^1^ Department of Sports Medicine, The First Affiliated Hospital, Guangdong Provincial Key Laboratory of Speed Capability, The Guangzhou Key Laboratory of Precision Orthopedics and Regenerative Medicine, Jinan University, Guangzhou, Guangdong, China; ^2^ The second Department of Orthopedics, Heyou Hospital, Foshan City, Guangdong, China; ^3^ College of Chemistry and Materials Science, Guangdong Provincial Key Laboratory of Spine and Spinal Cord Reconstruction, The Fifth Affiliated Hospital (Heyuan Shenhe People’s Hospital), Jinan University, Guangzhou, Guangdong, China; ^4^ Department of Rehabilitation Sciences, The Hong Kong Polytechnic University, Hong Kong, Hong Kong SAR, China; ^5^ Department of Orthopaedic, Huizhou First Hospital, Guangdong Medical University, Huizhou, Guangdong, China; ^6^ Institute of Future Health, School of Medicine, South China University of Technology, Guangzhou, Guangdong, China; ^7^ School of Medicine, Foshan University, Foshan, Guangdong, China

**Keywords:** tendon-bone healing, rotator cuff tear, mesoporous silica nanoparticles, dual-crosslinked hydrogels, tendon-bone interface (TBI), regeneration

## Abstract

**Introduction:**

During the healing process, the functional gradient attachment of the rotator cuff (RC) tendon-bone interface fails to regenerate, which severely impedes load transfer and stress dissipation, thereby increasing the risk of retears. As a result, the treatment of rotator cuff tears remains a significant clinical challenge.

**Methods:**

In this study, a dual-crosslinked hyaluronic acid/polyethylene glycol (HA/PEG) hydrogel scaffold was synthesized using hyaluronic acid and polyethylene glycol as base materials. Manganese-doped mesoporous silica nanoparticles (Mn-MSN) were incorporated into the hydrogel system to fabricate a manganese-based mesoporous silica nanoparticle-loaded dual-crosslinked hydrogel (Mn-MSN@Gel). The physicochemical properties of Mn-MSN@Gel, including porosity, elemental distribution, mechanical properties, biodegradability, and biocompatibility, were systematically characterized. The ion release profiles of Si^4+^ and Mn^4+^ were evaluated to assess sustained delivery. Rheological properties and self-healing capabilities were examined to determine injectability and in vivo stability. In vitro, the effects of Mn-MSN@Gel on cell migration, proliferation, and differentiation were assessed using rat bone marrow mesenchymal stem cells (rat-BMSCs) and tendon-derived stem cells (rat-TDSCs). The expression of osteogenic, tenogenic, oxidative stress-related, and inflammatory cytokine genes was analyzed. In vivo, a rat rotator cuff repair model was established to evaluate the biomechanical properties and tissue regeneration at the tendon-bone interface (TBI) following Mn-MSN@Gel injection.

**Results:**

Characterization demonstrated that Mn-MSN@Gel possesses a porous three-dimensional structure with uniform distribution of silicon, oxygen, and manganese elements, enabling sustained and slow release of Si^4+^ and Mn^4+^ ions. Additionally, the composite material exhibited excellent mechanical properties, biodegradability, and biocompatibility, while promoting cell migration/proliferation and accelerating regeneration of the tendon-bone interface. Mn-MSN@Gel enhanced the expression of osteogenic differentiation genes (Runx2, Alp, Sox9) in rat-BMSCs, upregulated tenogenic differentiation markers (Scx, Tnmd, Col3a1), and downregulated Mmp3 expression in rat-TDSCs. Furthermore, Mn-MSN@Gel modulated genes related to oxidative stress (Nrf2, Gpx4, Sod2) and inflammatory cytokines (IL-6, IL-10, Tnf-α), exhibiting anti-inflammatory effects and alleviating oxidative stress damage. In the rat rotator cuff repair model, Mn-MSN@Gel injection significantly improved postoperative biomechanical properties and promoted tissue regeneration at the TBI.

**Discussion:**

The self-healing and injectable properties of Mn-MSN@Gel ensure precise delivery and stable integration in vivo. By combining mechanical support with sustained release of bioactive ions, Mn-MSN@Gel provides a comprehensive therapeutic strategy for regenerative repair of the tendon-bone interface. Its biocompatibility and bioactivity facilitate cell recruitment, migration, and lineage-specific differentiation, which are crucial for reconstructing the functional gradient structure of the TBI. The anti-inflammatory and antioxidant effects further contribute to a favorable healing microenvironment. Overall, these findings indicate that Mn-MSN@Gel is a foundational biomaterial with significant therapeutic potential for enhancing structural regeneration and functional recovery of the TBI following rotator cuff injury.

## 1 Introduction

Rotator cuff tears (RCTs) represent one of the most prevalent tendon-bone injuries, frequently causing recurrent shoulder pain and restricted mobility. Statistical data indicate over 30 million annual global cases of tendon-bone interface (TBI) injuries (Maffulli et al., 2003), with more than 270,000 surgical procedures performed yearly in the United States alone for rotator cuff tendon repair (Saveh-Shemshaki and Laurencin, 2019). The TBI constitutes a structurally intricate and functionally essential transitional zone connecting tendon to bone, crucial for maintaining shoulder joint integrity. Despite advancements in surgical techniques for rotator cuff repair, RC prosthetic surgery failure rates remain elevated (20%–94%) (Galatz et al., 2004; Xu et al., 2021) due to inadequate bone-tendon healing and regeneration. This clinical challenge arises from the non-regeneration of the rotator cuff functionally graded tendon-bone attachment during postoperative healing, which fails to reconstruct the TBI’s physiological layered architecture (tendon, uncalcified fibrocartilage, calcified fibrocartilage, and bone). Instead, fibrovascular scar tissue with inferior mechanical properties forms, substantially compromising load transfer efficiency and stress dissipation capacity (Zumstein et al., 2017), thereby increasing retear susceptibility. Recent years have witnessed growing interest in tissue engineering material applications for TBI treatment. Numerous material-based strategies have been developed to enhance TBI healing, demonstrating potential for functional reintegration of injured rotator cuffs (Zhao et al., 2014a; Wang et al., 2023; Zhu et al., 2021). Nevertheless, achieving functional regeneration of native tendon enthesis at the tendon-bone interface persists as a significant clinical challenge.

Optimal tendon-bone interface (TBI) healing is fundamentally determined by vascularization and osteogenesis (Zhao et al., 2022), with healing strength contingent upon bone tissue ingrowth, mineralization, and maturation (Rodeo et al., 1993). The regeneration extent of fibrocartilaginous zones and osseous regions critically influences clinical outcomes in injured patients (Xu et al., 2021; Shengnan et al., 2021). Rodeo et al. (1999) demonstrated that bone morphogenetic protein (BMP) treatment enhances peri-tendinous bone formation, improves bone-tendon integration, and increases biomechanical strength, thereby accelerating TBI healing. Calcium phosphate, recognized for its osteogenic properties, has been extensively employed to augment bone regeneration. Enhanced osteogenic differentiation in rat bone marrow mesenchymal stem cells (BMSCs) manifests through upregulated expression of Rux2, ALP, and OPN markers (Ren et al., 2023). Liao et al. (2021) reported histological improvements in bone tunnels using calcium phosphate-treated tendon grafts during goat anterior cruciate ligament (ACL) reconstruction, facilitating TBI healing. Innovative approaches utilizing growth factor-loaded nano-scaffolds have shown promise. Zhao et al. (2014b) developed basic fibroblast growth factor (bFGF)-incorporated electrospun poly (lactic-co-glycolic acid) (PLGA) membranes for rotator cuff repair, demonstrating enhanced cell adhesion/proliferation and accelerated tendon-bone reconstruction. Inflammatory responses and oxidative stress at the bone-tendon junction significantly impact repair outcomes. Inflammation critically influences tissue healing responses to transplants and medical devices. Connizzo and Grodzinsky (2018) revealed that pro-inflammatory factors in murine TBI models downregulate tenocyte markers (Scx, Tnmd, Col3α1) and induce cell death. Key inflammatory components in tendon repair include IL-6, IL-8, Tnf-α, MMP-1, and MMP-3 (Jensen et al., 2018), with MMP-1/3/13 exhibiting collagen/proteoglycan degradation capacity (Nissinen and Kähäri, 2014), potentially impairing TBI healing. IL-6, IL-1β, and Tnf-α stimulate receptor activator of nuclear factor κB ligand (RANKL) expression in synovial fibroblasts/osteoblasts, promoting osteoclastogenesis (Mori et al., 2011). IL-6 additionally inhibits synovial fibroblast proliferation while upregulating TIMP-1 in synovial/chondrocytic cells (Mori et al., 2011). Nikhil et al. (Oak et al., 2014) demonstrated that 5-LOX and COX-1/2 inhibition enhances post-repair fibrosis and adipogenesis in RCT models. Oxidative stress during healing modulates tendon stem cell apoptosis/autophagy (Li et al., 2020), with Ren et al. (2023) confirming that antioxidant intervention at injury sites promotes tendon regeneration. Upregulated SOD1/SOD2 expression enhanced TBI healing in rat RCT models, while the AMPK/Nrf2/GPX4 pathway critically regulates tenocyte apoptosis/senescence (Chen et al., 2024). Collectively, these findings underscore that coordinated osteochondral differentiation at TBI interfaces coupled with inflammatory/oxidative stress modulation represents a promising therapeutic strategy for bone-tendon integration.

It is well-known that the primary factors determining perfect healing of the tendon-bone interface are vascular and bone formation (Zhao et al., 2022). The healing strength of the tendon-bone interface is determined by the ingrowth, mineralization, and maturation of bone tissue (Rodeo et al., 1993). The degree of regeneration in the fibrocartilaginous zone and bone tissue area plays a crucial role in satisfying clinical outcomes for injured patients (Xu et al., 2021; Shengnan et al., 2021). Scott A and colleagues (Rodeo et al., 1999) found that treatment with Bone morphogenetic protein (BMP) could lead to more extensive bone formation around the tendon, tighter integration between the new bone and the tendon, and higher biomechanical strength, significantly promoting tendon-bone healing. Additionally, calcium phosphate, known for its bone formation-promoting advantages, has been widely used to enhance bone regeneration. Promoting bone regeneration of rat BMSCs can be manifested as increased expression of osteogenic markers Rux2, ALP, and OPN (Ren et al., 2023). Researchers have also experimented with growth factor-loaded nano scaffolds to promote tendon-bone healing. Zhao et al. (2014b) developed an electrospun polylactic acid-glycolic acid (PLGE) fiber membrane loaded with basic fibroblast growth factor (bFGF) for rotator cuff repair, showing that the product facilitated cell attachment and proliferation, accelerating tendon-bone reconstruction. Inflammation and oxidative stress at the bone-tendon junction also play a crucial role in repair outcomes. Inflammation is a key factor in determining tissue healing outcomes and responses to transplants and other medical devices. For example, the study by Brianne K et al. found that in a mouse tendon-bone injury model, pro-inflammatory factors released by muscles and bones can lead to a decrease in the expression of tendon cell markers Scx, Tnmd, and Col3a1, and even cause cell death (Connizzo and Grodzinsky, 2018). Regarding tendon injury and repair, many inflammation-associated mediators and cell types have been identified, such as IL-6, IL-8, TNF-α, matrix metalloproteinase (MMP)-1, MMP-3, etc. (Jensen et al., 2018); MMP-1, MMP-3, and MMP-13 can degrade proteins such as types 1, 2, and 3 collagen and proteoglycans (Nissinen and Kähäri, 2014), which may not be good news for TBI healing. IL-6, IL-1, and tumor necrosis factor have also been shown to stimulate RANKL expression on synovial fibroblasts and osteoblasts, leading to osteoclastogenesis (Mori et al., 2011). IL-6 can inhibit the proliferation of synovial fibroblasts and stimulate the expression of MMP 1 tissue inhibitor (TIMP-1) in synovial cells and chondrocytes (Mori et al., 2011). Nikhil R and others reported that inhibiting inflammatory factors 5-LOX, COX-1, and COX-2 can increase fibrosis and fat formation after rotator cuff injury repair (Oak et al., 2014); furthermore, during the injury repair process, oxidative stress and reactive oxygen species can affect the apoptosis and autophagy of tendon stem cells (Li et al., 2020). Research by Xunshan R and others (Ren et al., 2023) has proven that counteracting oxidative stress damage at the site of tendon injury can effectively promote tendon regeneration. Enhanced expression of antioxidant enzymes such as SOD1 and SOD2 promoted tendon-bone healing after rotator cuff repair in rats. In addition, the oxidative stress signaling pathway AMPK/Nrf2/GPX4 also plays a crucial role in tendon cell apoptosis and senescence (Chen et al., 2024). In summary, promoting bone and cartilage differentiation at the TBI interface, and regulating the inflammatory and oxidative stress in the microenvironment, will be effective therapeutic means to promote tendon-bone healing.

This study employed hyaluronic acid (HA) and polyethylene glycol (PEG) as bioactive substrates to fabricate an adhesive hydrogel system via click chemistry, combining biological functionality with enhanced mechanical properties and handling characteristics. The hydrogel system enables rapid *in situ* adhesion at injury sites post-injection, providing a three-dimensional scaffold that facilitates cellular attachment and proliferation, thereby accelerating tissue repair. To optimize mechanical performance and nano-structural modification, manganese-doped mesoporous silica nanoparticles (Mn-MSN) were incorporated into the HA/PEG hydrogel matrix, yielding manganese-based mesoporous silica nanoparticle-incorporated dual-crosslinked hydrogels (Mn-MSN@gel). Characterization revealed that Mn-MSN@gel possesses a porous three-dimensional architecture with homogeneous distribution of Si, O, and Mn elements. The composite material demonstrated favorable mechanical strength, injectability, self-healing capability, biodegradability, and biocompatibility. Mn-MSN@gel enables controlled Mn^4+^ release, exhibiting anti-inflammatory effects through IL-6 and TNF-α suppression coupled with IL-10 elevation, while mitigating oxidative stress via Nrf2, GPX4, and SOD2 activation. The hydrogel promoted osteogenic differentiation in rat bone marrow mesenchymal stem cells (BMSCs), evidenced by upregulated Rux2, ALP, and Sox9 expression, and enhanced tenogenic differentiation in tendon-derived stem cells (TDSCs) through increased Scx, Tnmd, Col3α1, and MMP3 markers. In rat rotator cuff repair models, Mn-MSN@gel significantly improved biomechanical properties of regenerated tissue and facilitated tendon-bone interface (TBI) reconstruction. These findings collectively demonstrate Mn-MSN@gel’s potential as a foundational biomaterial for TBI repair in rotator cuff injuries.

## 2 Materials and methods

### 2.1 Reagents

Cetyltrimethylammonium Tosylate (CTATos), Triethanolamine (TEAH3), Tetraethyl Orthosilicate (TEOS), and Potassium Permanganate (KMnO_4_) were purchased from Shanghai Mclean Biochemical Technology Co., Ltd (China). Trizol reagent was purchased from Beyotime Technology Co., Ltd. (China), Lipopolysaccharide (LPS), Phosphate Buffered Saline (PBS), Penicillin/Streptomycin (P/S), and Fetal Bovine Serum (FBS) were purchased from Gbico (USA). The CCK-8 assay kit was purchased from Dojindo Laboratories (Tokyo, Japan).

### 2.2 Synthesis of HA/PLG dual crosslinked hydrogel (gel)

Dual-crosslinked hydrogels were synthesized via a two-step orthogonal crosslinking strategy. First, HA-furan-CHO and HA-furan-NH_2_ solutions were mixed with maleimide-functionalized PEG (mal-PEG-mal) crosslinker. The primary network formed immediately through Schiff base reactions between amine (-NH_2_) and aldehyde (-CHO) groups. Subsequently, a secondary network was established via Diels-Alder click chemistry between furan and maleimide groups at room temperature. This sequential approach enabled precise architectural control of the hydrogel (designated as “Gel” in subsequent experiments) through orthogonal chemical bonding mechanisms.

### 2.3 Synthesis of Mn-MSN@gel

MSN was prepared according to the method previously reported (Xiong et al., 2023). 0.5 g of MSN powder was added to 100 mL of 1M KMnO_4_ solution and magnetically stirred in a 40°C water bath for 6 h. After the reaction, the precipitate was collected by centrifugation, washed three times with deionized water, freeze-dried, and the freeze-dried powder was calcined in a muffle furnace at 510°C for 6 h to obtain black Mn-MSN powder. The modified hyaluronic acid was prepared into 8% solutions, and different concentrations (0%, 0.4%, 0.6%, 0.8%, 1.0%) of Mn-MSN-NH_2_, Mn-MSN-COOH, and Mn-MSN were mixed and added into a polytetrafluoroethylene mold. After the hydrogel was formed, it was removed, resulting in Mn-MSN@gel.

### 2.4 Characterization of MSN and Mn-MSN@gel

The phases of Gel, MSN, and Mn-MSN were identified using ^1^H-NMR spectroscopy (XRD; Miniflex 600, Rigaku, Japan), X-ray diffraction (XRD; Miniflex 600, Rigaku, Japan), and Fourier-transform infrared spectroscopy (FTIR; Nicolet is10, Thermo Scientific, USA). The morphology and structure of MSN and Mn-MSN were observed using field emission scanning electron microscopy (FESEM; Ultra 55, Zeiss, Germany) and high-resolution transmission electron microscopy (HRTEM; JEM-2100F, JEOL, Japan). The particle size and zeta potential of MSN, Mn-MSN, and Mn-MSN@gel were measured using a particle analyzer (Nano ZS, Malvern, USA). The content of Mn and Si in MSN and Mn-MSN was analyzed using inductively coupled plasma atomic emission spectroscopy (ICP-AES; Optima 5300DV, PerkinElmer, USA). The surface area and pore structure of MSN, Mn-MSN, and Mn-MSN@gel were measured using an analyzer (3H-2000PS2, Beishide Instrument, China). For each batch, randomly select a subset of samples for identical quality inspections and scans to ensure consistent and dependable material quality.

### 2.5 Cell extraction and cultivation

Rat-BMSCs were isolated from 6-week-old Sprague-Dawley rats femurs as previously literature (Lennon and Caplan, 2006). Rat-TDSCs were taken from the Achilles tendon, as previously reported (Liu et al., 2018). Cells were cultured in an environment at 37°C, with 5% CO2 concentration and 95% relative humidity, and the culture medium was changed every 2 days. Adherent BMSCs and TDSCs were digested with trypsin and resuspended for subsequent experiments.

### 2.6 Cytotoxicity detection

BMSCs were seeded at 2 × 10^4^ cells per well in a 12-well plate and allowed to adhere for 12 h. Gel and Mn-MSN@gel were then added to the cell chambers for co-culture with BMSCs for 1, 3, and 5 days. The cell viability was calculated strictly according to the CCK-8 Cell Viability Assay Kit manual, with each sample tested in triplicate. Live/Dead Staining: PI-AM live/dead double staining solution was added and incubated in the dark for 30 min, followed by washing three times with PBS solution, and images were taken under an inverted fluorescence microscope.

### 2.7 Alkaline phosphatase assay

BMSCs were seeded at 5 × 10^4^ cells per well in a 12-well plate and allowed to adhere for 12 h. PBS (control group), Gel, and Mn-MSN@gel were added to the cell chambers. The culture medium was switched to osteogenic induction medium, and cells were co-cultured for 7 and 14 days. Cells were washed three times with PBS, fixed with 4% paraformaldehyde for 30 min, stained with BCIP/NBT kit, and observed and photographed under an inverted fluorescence microscope.

### 2.8 Calcium nodule staining

BMSCs were seeded at 5 × 10^4^ cells per well in a 12-well plate and allowed to adhere for 12 h. After switching to osteogenic induction medium and co-culturing with BMSCs for 14 and 21 days, the osteogenic induction medium was removed from the cell chambers, and the cells were washed three times with PBS. Cells were fixed with 500 μL of 4% paraformaldehyde for 30 min, the paraformaldehyde was removed, and cells were washed three times with PBS. Cells were then stained with 1% Alizarin Red for 30 min, the staining solution was removed, and cells were washed with PBS. Observations and photographs were taken under an inverted fluorescence microscope.

### 2.9 Quantitative reverse transcription polymerase chain reaction (RT-PCR)

Rat BMSCs and TDSCs were seeded at a density of 2 × 10^4^ cells per well in a 6-well plate, cultured for 24 h, and treated with 10 μg/mL LPS medium for 12 h to simulate inflammation and injury repair in tendon cells. In the NC (PBS as control) group, Gel group, and Mn-MSN@gel group, total RNA was extracted from BMSCs and TDSCs samples using an RNA extraction kit (Catalog No. 74106, Qiagen, Hilden, Germany). Subsequently, the RNA was reverse transcribed into cDNA using a reverse transcription kit (Catalog No. 205413, Qiagen). The resulting cDNA samples were then analyzed via real-time polymerase chain reaction using a SYBR Green PCR kit (Catalog No. 208056, Qiagen). The amplification process included an initial incubation at 95°C for 2 min, followed by 40 cycles of 95°C for 5 s for denaturation and 60°C for 15 s for annealing. A melting curve analysis was performed to ensure the specificity of the amplification. Primers for the target genes (listed in Table 1) were used. The expression levels of the target genes were normalized to the housekeeping gene GAPDH. Calculations were performed using the 2^−ΔΔCT^ method.

**TABLE 1:** Primer sequences used for RT- PCR.

Gene name	Forward (5′→3′)	Reverse (5′→3′)
Sox9	AGA​GCG​TTG​CTC​GGA​ACT​GT	TCC​TGG​ACC​GAA​ACT​GGT​AAA
Rux2	CCGATGGGACCGTGGTT	CAG​CAG​AGG​CAT​TTC​GTA​GCT
Alp	GCA​CAA​CAT​CAA​GGA​CAT​CG	TCA​GTT​CTG​TTC​TTG​GGG​TAC​AT
Scx	AAC​ACG​GCC​TTC​ACT​GCG​CTG	CAG​TAG​CAC​GTT​GCC​CAG​GTG
Tnmd	GTG​GTC​CCA​CAA​GTG​AAG​GT	GTC​TTC​CTC​GCT​TGC​TTG​TC
Col3a1	CTT​CTC​ACC​CTG​CTT​CAC​CC	GGG​CAG​TCT​AGT​GGC​TCA​TC
Mmp3	AGA​CAA​AGA​GTT​GGC​AGT​GCA​AT	CTG​TAT​GTG​ATC​TGG​TTC​TTG​TCC​C
Nrf2	GAC​AAA​CAT​TCA​AGC​CGA​TTA​GAG​G	ACT​TTA​TTC​TTC​CCT​CTC​CTG​CGT
Gpx4	AAG​TAC​AGG​GGT​TGC​GTG​TG	GGG​CAT​CGT​CCC​CAT​TTA​CA
Sod2	TCA​TGC​AGC​TGC​ACC​ACA​GC	CCA​TTG​AAC​TTC​AGT​GCA​GG
IL-6	CCA​ACT​TCC​AAT​GCT​CTC​CTA​AT	CGA​GTA​GAC​CTC​ATA​GTG​ACC​TT
IL-10	ATG​GAG​GAG​CGA​AGG​TTA​GTG​GTC	ACT​CTT​GTT​CTC​ACA​CGG​CAG
Tnf-α	CCA​GAC​CCT​CAC​ACT​CAG​ATC​AT	CGG​CAG​AGA​GGA​GGT​TGA​CT

### 2.10 Rotator cuff injury model

Male SD rats aged 8 weeks were used to establish a tendon injury model (n = 5). The rat rotator cuff injury model was created in accordance with methods previously reported (Chen W. et al., 2021). Subsequently, 100 μL of Gel and Mn-MSN@Gel were injected into the surgical area using a 2 mL syringe (the control group received an injection of 100 μL saline).

### 2.11 Biomechanical testing

When collecting samples, the supraspinatus muscle was preserved intact at the TBI site connecting to the humeral head. Carefully remove the connective and adipose tissue around the supraspinatus tendon, measure the width and thickness of the tendon with calipers, and calculate its cross-sectional area using the elliptical formula. Mount the specimen on an electronic universal testing machine and pull the tendon at a speed of 20 mm/min until TBI rupture. Record changes in load values, ultimate stress, and plot the stress-strain curve throughout the process. Calculate tensile strength (ultimate stress/cross-sectional area) and elastic modulus (calculated from the slope of the tangent at the initial point of the stress-strain curve).

### 2.12 Histological staining and scoring

After all samples were collected, three samples from each group were randomly selected for decalcification, fixation, and then embedded in paraffin in the coronal position, and sectioned to a thickness of about 5 μm. Sections were stained with hematoxylin and eosin (H&E), Safranin O-Fast Green, Masson’s trichrome, and toluidine blue for histological analysis. The degree of fiber maturity and cartilage repair at the repaired tendon-bone interface was evaluated. H&E sections were scored based on the density of neofibers, fiber parallelism, fiber density, and TBI maturity in the TBI area (Table 2) to assess the repair of the tendon-bone interface.

**TABLE 2:** Scoring table for tendon-bone interface repair.

Score	0	1	2	3
Newly formed fiber density	0%–25%	25%–50%	50%–75%	75%–100%
Fiber parallelism	0%–25%	25%–50%	50%–75%	75%–100%
Fiber density	0%–25%	25%–50%	50%–75%	75%–100%
tendon-Bone interface maturity	0%–25%	25%–50%	50%–75%	75%–100%

Scoring criteria: The ratio of the area occupied by the above indicators to the area of the tendon-bone interface.

### 2.13 Statistical analysis

Statistical graphs were created using GraphPad Prism 8.0 software (GraphPad software, San Diego, California, USA). Data analysis was conducted using SPSS software for Windows (Version 20.0; SPSS, Inc., IL, USA). To compare multiple groups, one-way analysis of variance was performed, followed by Fisher’s least significant difference (LSD) test for multiple comparisons, or Dunnett’s test when variances were not equal among all groups. When comparing two groups, a two-tailed Student’s t-test was used. Data are expressed as mean ± SD, and *p < 0.05 was considered statistically significant.

## 3 Results

### 3.1 Physicochemical characterization of MSN and Mn-MSN

**FIGURE 1:**
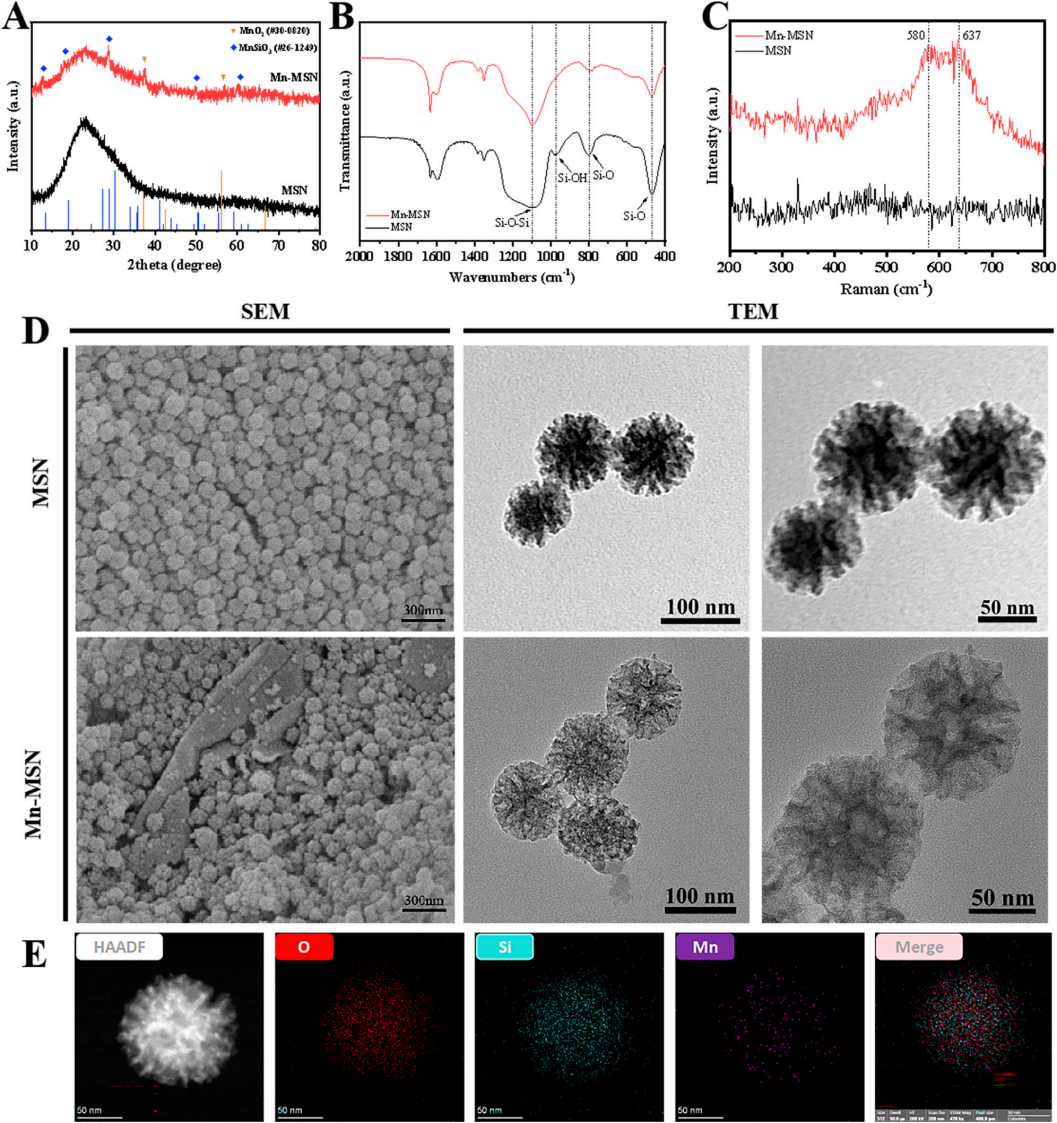
Characterization of MSN and Mn-MSN; **(A)** XRD spectrum; **(B)** FTIR spectrum, **(C)** Raman spectrum **(D)** SEM images and TEM images; **(E)** EDS spectrum of Mn-MSN.

Figure 1A displays the XRD patterns of MSN and Mn-MSN. MSN exhibited a broad diffraction peak at 15°–35°, confirming its amorphous silica structure. In contrast, Mn-MSN demonstrated distinct crystalline peaks corresponding to manganese dioxide (MnO_2_) and manganese silicate (MnSiO_3_), confirming the coexistence of both phases. As illustrated in Figure 1B, FTIR analysis revealed characteristic absorption bands: the Si-O-Si asymmetric stretching vibration at 1091 cm^−1^, Si-OH bending vibration at 978 cm^−1^, and Si-O stretching vibrations at 802 cm^−1^ and 469 cm^−1^. The broad peak at 1630 cm^−1^ corresponded to adsorbed water molecules, verifying the predominant SiO_2_ composition in both materials. Figure 1C presents Raman spectra showing Mn-O vibrational bands at 580 cm^−1^ and 637 cm^−1^ in Mn-MSN, confirming Mn-O bonding (Han et al., 2020). SEM and TEM images in Figure 1D revealed spherical morphology with uniform particle sizes (∼100 nm) for both MSN and Mn-MSN. No significant morphological alterations were observed after MnO_2_ formation, indicating preserved surface characteristics. TEM analysis further confirmed the maintenance of dendritic mesoporous structures in Mn-MSN, demonstrating structural integrity post-modification. Figure 1E shows EDS elemental mapping of Mn-MSN, revealing homogeneous spatial distribution of Si, O, and Mn throughout the matrix. This confirms successful *in situ* generation of MnO_2_ within MSN mesopores.

**FIGURE 2:**
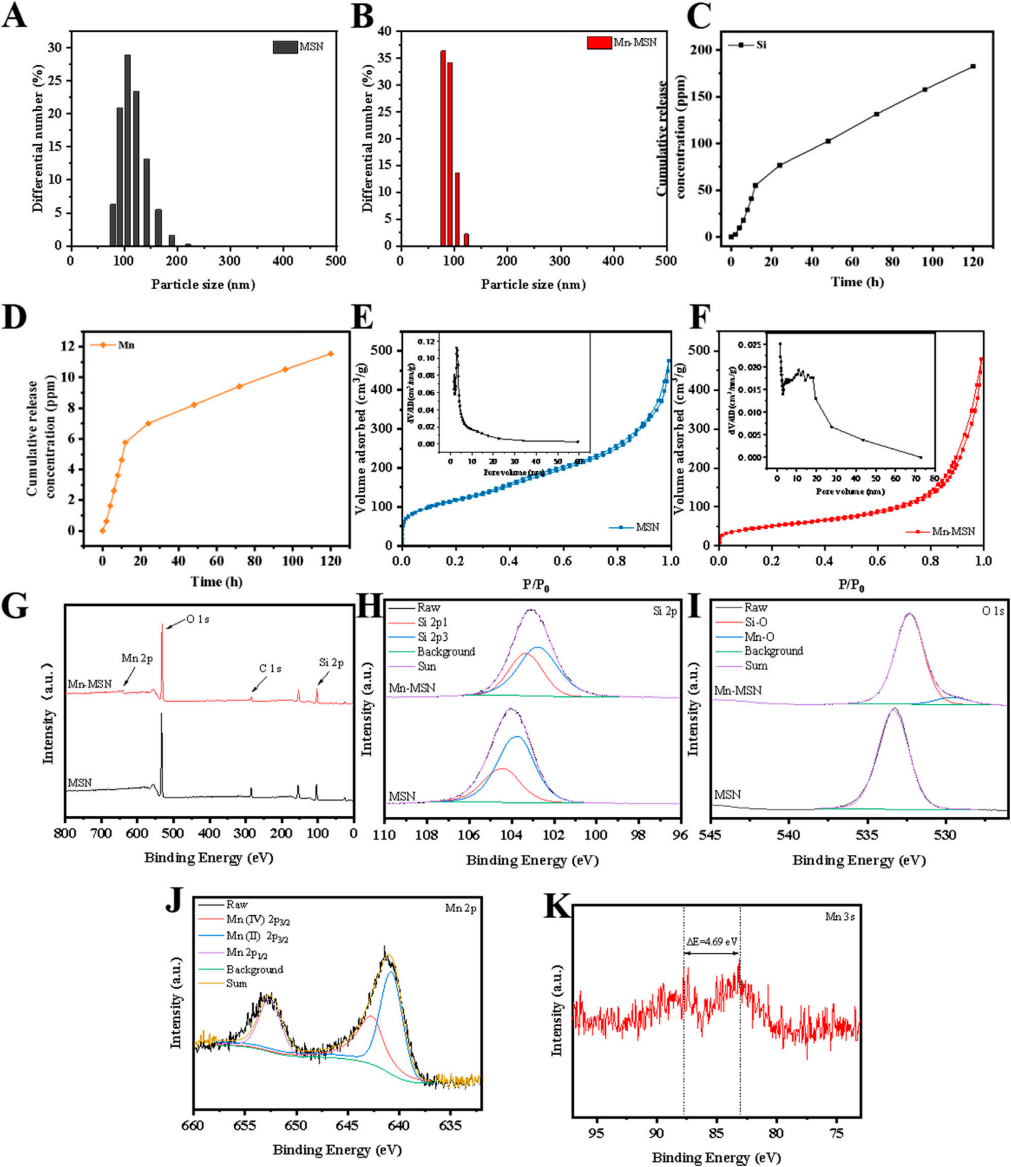
**(A,B)** Particle size distribution diagrams; **(C,D)** Mn-MSN ion cumulative release curves, **(E,F)** N2 adsorption-desorption isotherms; **(G)** XPS spectra. Full spectrum, **(H)** Si 2p high resolution spectrum, **(I)** O 1s high resolution spectrum, **(J)** Mn 2p high resolution spectrum, **(K)** Mn 3s high resolution spectrum.

Figures 2A,B demonstrate that the particle size of MSN is predominantly distributed around 122.4 nm with a relatively broad size distribution. In contrast, Mn-MSN exhibits reduced particle sizes centered at approximately 78.8 nm and displays a narrower size distribution. Figures 2C,D reveal substantial release of Si^4+^ and Mn^4+^ during the initial 20 h, followed by a gradual decrease in cumulative release rates. Notably, the cumulative Si^4+^ release significantly exceeds that of Mn^4+^. Figures 2E,F present type IV N_2_ adsorption-desorption isotherms for both MSN and Mn-MSN, characteristic of mesoporous materials. The continuous upward trend at high P/P_0_ values indicates multilayer N_2_ adsorption, while the H1-type hysteresis loop confirms the preservation of mesoporous structures after MnO_2_ *in-situ* generation.

The XPS survey spectrum (Figure 2G) identifies Si and O elements in MSN, with additional Mn detected in Mn-MSN. High-resolution Si 2p spectra (Figure 2H) show binding energy shifts from 103.74 eV (MSN) to 102.77 eV (Mn-MSN), accompanied by a characteristic SiO_2_ peak at 103.5 eV. O 1s spectra (Figure 2I) display a single peak at 533.32 eV for MSN, while Mn-MSN exhibits dual peaks at 532.26 eV (Si-O) and 529.42 eV (Mn-O). Mn 2p spectra (Figure 2J) reveal characteristic peaks at 652.62 eV (Mn 2p_1_/_2_), 642.74 eV (Mn^4+^ 2p_3_/_2_), and 640.79 eV (Mn^2+^ 2p_3_/_2_), confirming the coexistence of Mn^4+^ and Mn^2+^. The Mn 3s spectrum (Figure 2K) displays a distinctive doublet structure arising from 3s-3d electron coupling, where the splitting energy (ΔE) correlates with manganese oxidation states: ΔE = 6.0 eV (Mn^2+^ in MnO), ΔE ≥5.3 eV (Mn^3+^ in Mn_2_O_3_), and ΔE = 4.7 eV (Mn^4+^ in MnO_2_).

### 3.2 Mechanical and self-healing properties of Mn-MSN@Gel nanocomposite hydrogel

**FIGURE 3:**
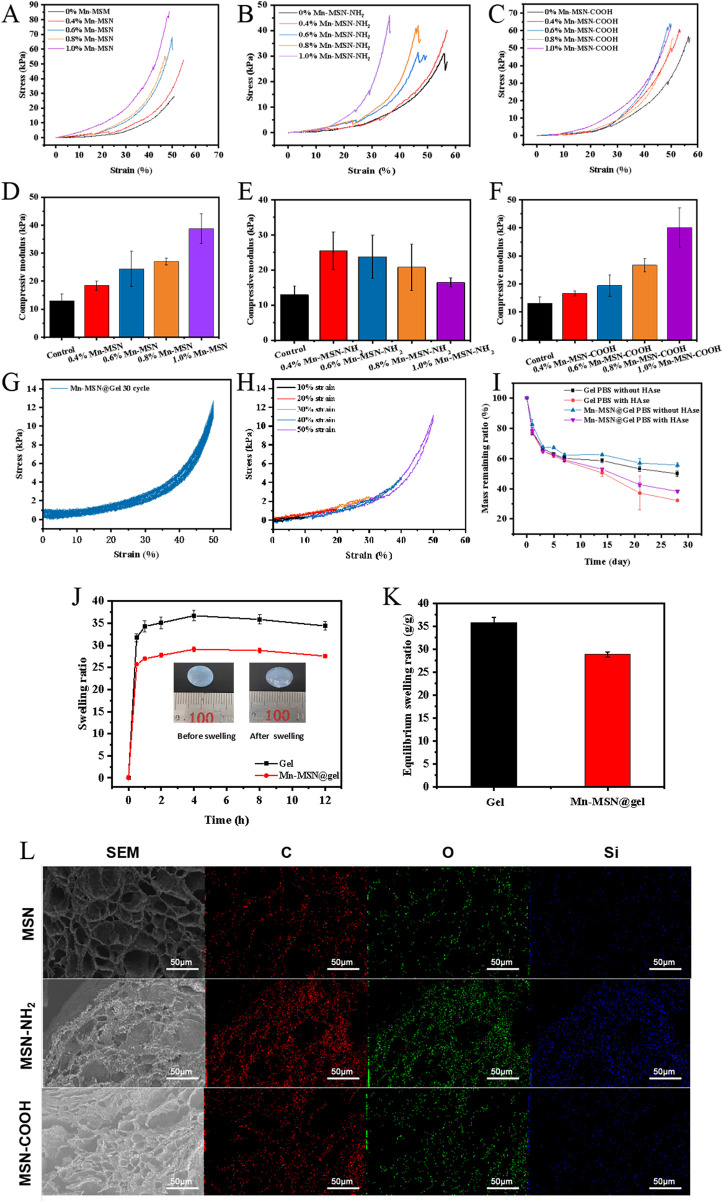
Three different Mn-MSN@Gel nanocomposite hydrogels; **(A–C)** Stress-strain curves; **(D–F)** compressive modulus; **(G)** Cyclic compressive loading-unloading curves of hydrogels at 50% strain; **(H)** Compressive loading-unloading curves of hydrogels under different strains (10%, 20%, 30%, 40% and 50%); **(I)** Degradation performance; **(J)** Swelling curves; **(K)** Equilibrium swelling ratios of three different Mn-MSN@Gel nanocomposite hydrogels; **(L)** Cross-sectional micro-morphology.

Figures 3A–C demonstrate that increasing concentrations of Mn-MSN, Mn-MSN-NH_2_, and Mn-MSN-COOH progressively reduce hydrogel deformation under equivalent stress conditions, indicating enhanced anti-deformation capacity in nanocomposite hydrogels. Figures 3D–F reveal distinct concentration-dependent mechanical responses: the compressive modulus increases from 18.46 ± 1.67 kPa to 38.69 ± 5.31 kPa for Mn-MSN (0.4%–1%) and from 16.63 ± 0.90 kPa to 40.09 ± 7.01 kPa for Mn-MSN-COOH, while decreasing from 25.51 ± 5.33 kPa to 16.47 ± 1.33 kPa for Mn-MSN-NH_2_. Based on these mechanical performance evaluations and nanoparticle synthesis considerations, 1% Mn-MSN was selected as the optimal inorganic filler for preparing Mn-MSN@Gel nanocomposite hydrogel.

Cyclic compression testing (50% strain, 30 cycles) demonstrates excellent fatigue resistance in Mn-MSN@Gel, evidenced by overlapping loading-unloading curves and minimal stress reduction (Figure 3G). The observed hysteresis loops originate from internal energy dissipation mechanisms. Figure 3H displays consistent stress-strain curve profiles across incremental strains (10%–50%), with no significant hysteresis, confirming superior compression recovery performance. These mechanical characteristics position Mn-MSN@Gel as a promising material for applications requiring durable elastic properties.

Figure 3I illustrates the degradation profiles of Mn-MSN@Gel hydrogel in PBS and PBS containing 150 U/mL hyaluronidase (HAse). The Mn-MSN@Gel group exhibited slower degradation kinetics compared to pure Gel hydrogel, with residual mass percentages after 28 days measuring 55.56% ± 1.54% (PBS) and 38.27% ± 0.99% (PBS/HAse) versus 49.89% ± 1.86% and 32.21% ± 0.80% for the control group, respectively. These results confirm that 1% Mn-MSN incorporation effectively modulates hydrogel degradation rates. Figures 3J,K display swelling kinetics and equilibrium swelling ratios, revealing rapid initial absorption followed by stabilization at 4 h. The Mn-MSN@Gel nanocomposite demonstrated reduced swelling capacity (28.84 ± 0.60 g/g) compared to pure Gel hydrogel (35.85 ± 1.06 g/g). Figure 3L presents microstructural characterization showing preserved porous architecture in MSN-containing hydrogels. Energy-dispersive X-ray spectroscopy (EDS) mapping confirmed homogeneous distribution of Si and O elements within the hydrogel matrix, verifying uniform dispersion of MSN, MSN-NH_2_, and MSN-COOH nanoparticles.

**FIGURE 4:**
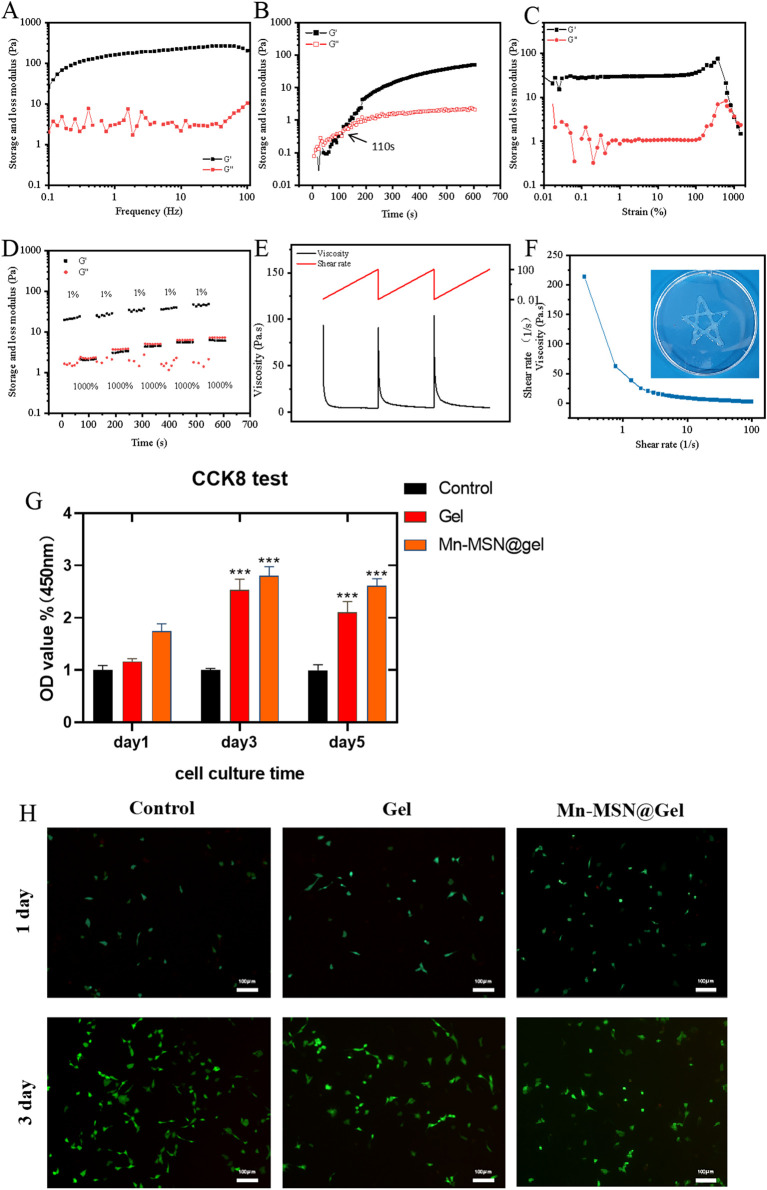
**(A)** Storage modulus and loss modulus, **(B)** Time scan, **(C)** Strain scan **(D)** alternating step strain scan, **(E–F)** shear thinning properties and injectability of nanocomposite hydrogels, **(G)** CCk8 cytotoxicity detection; **(H)** BMSCs live/dead staining image; (*, P < 0.05; **, P < 0.01; ***, P < 0.001).

Figure 4A demonstrates frequency-independent viscoelastic behavior of the Mn-MSN@Gel nanocomposite hydrogel, where storage modulus (G′) consistently exceeds loss modulus (G″) across the tested frequency range, confirming solid-like characteristics with preserved structural integrity. Figure 4B reveals a 110-second gelation time for Mn-MSN@Gel, marginally prolonged compared to 84 s for the pure Gel formulation. Strain amplitude testing (Figure 4C) identifies a critical strain threshold at 1000%, below which G′ > G″ confirms maintained gel integrity, while exceeding this limit induces structural collapse (G″ > G′). Cyclic strain testing (Figure 4D) demonstrates remarkable self-healing capacity through five consecutive cycles of high (1000%) and low (1%) strain alternation, showing complete modulus recovery (G′ > G″) after each structural disruption. The hydrogel exhibits pseudoplastic behavior (Figures 4E,F) with reversible viscosity modulation from 1,200 Pa·s (0.1 s^−1^) to 20 Pa·s (100 s^−1^), enabling smooth injection through 18-gauge needles while maintaining structural reconstitution capacity. These rheological analyses confirm the preservation of intrinsic hydrogel properties (self-healing capacity, shear-thinning behavior) following Mn-MSN incorporation, with enhanced mechanical stability through nanoparticle reinforcement.

### 3.3 Cell compatibility detection of gel and Mn-MSN@Gel

Figure 4G presents biocompatibility assessments through BMSC proliferation assays. Both Gel and Mn-MSN@Gel hydrogels demonstrated cytocompatibility, showing no significant differences in cell viability compared to controls after 24-h culture. Notably, prolonged culture revealed enhanced proliferative effects: at day 3, Mn-MSN@Gel exhibited 2.8 ± 0.17-fold (***P < 0.001) and Gel showed 2.5 ± 0.21-fold (***P < 0.001) increases in metabolic activity relative to control. This trend persisted through day 5, with Mn-MSN@Gel maintaining superior proliferation (2.6 ± 0.13-fold, ***P < 0.001) compared to Gel (2.1 ± 0.21-fold, ***P < 0.001), suggesting nanoparticle-mediated enhancement of bioactivity. Live/dead staining analysis (Figure 4H) confirmed preserved cellular morphology, with BMSCs maintaining characteristic spindle shapes and comparable densities across hydrogel and control groups throughout the 3-day observation period. Quantitative cell counts revealed >90% viability in all conditions, with no significant morphological alterations. These collective findings demonstrate that Mn-MSN incorporation not only preserves but enhances the hydrogel’s inherent biocompatibility while promoting stem cell proliferation–critical attributes for biomedical applications requiring cell-material interactions.

### 3.4 Cell osteogenic differentiation performance of gel and Mn-MSN@Gel

**FIGURE 5:**
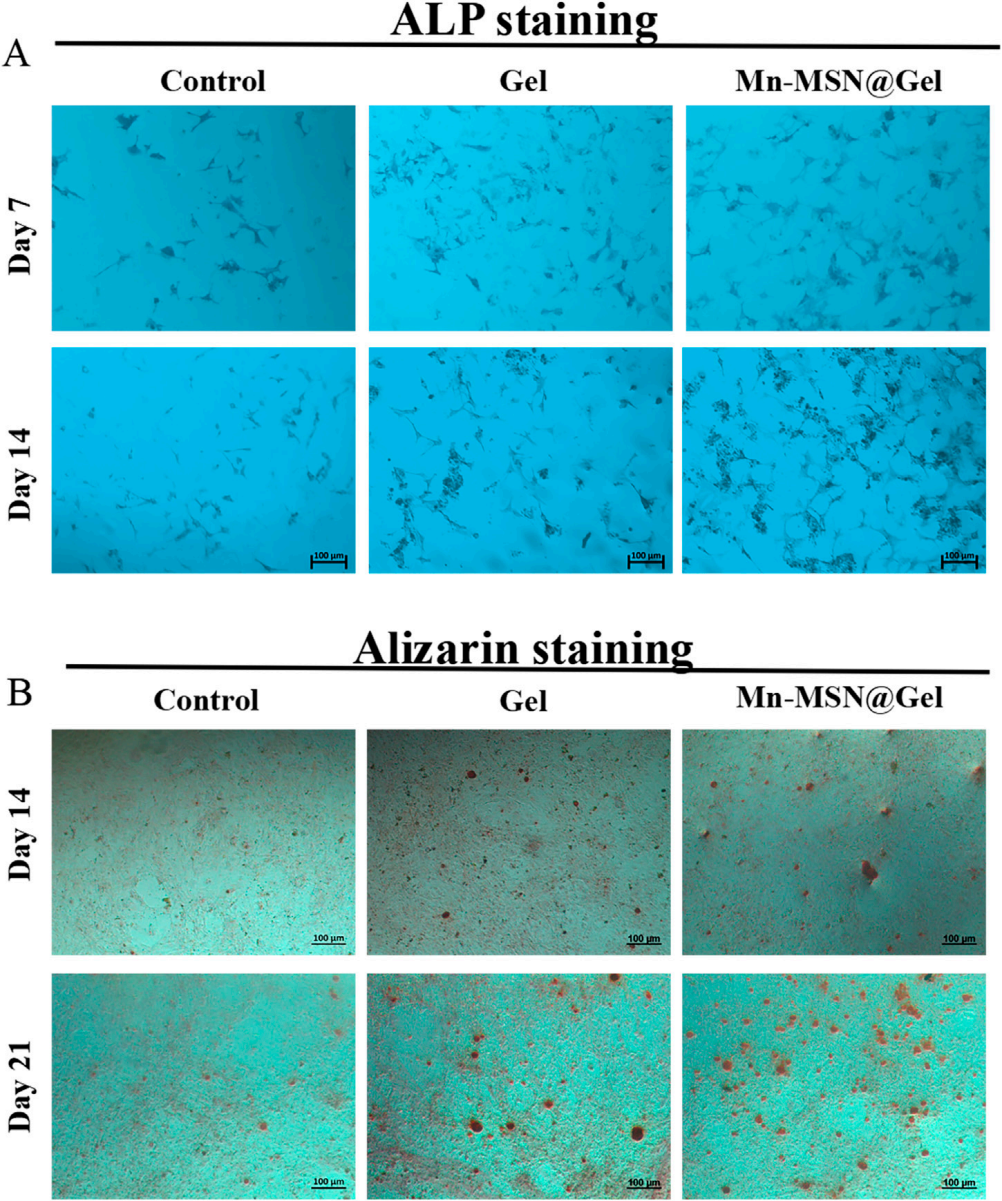
**(A)** Qualitative analysis of ALP for 7 days and 14 days of co-culture of BMSCs, **(B)** Qualitative analysis of calcium nodules at 14 and 21 days.

Alkaline phosphatase (ALP), a hallmark enzyme of mature osteogenic differentiation in BMSCs, exhibits staining intensity proportional to its activity, reflecting osteogenic differentiation capacity. The osteogenic potential of Mn-MSN@Gel hydrogel was assessed via ALP and Alizarin Red S assays (He et al., 2024). Figure 5A demonstrates that both Gel and Mn-MSN@Gel groups displayed intensified ALP staining compared to the Control group after 7- and 14-day cultures, with Mn-MSN@Gel exhibiting the most pronounced coloration, confirming superior ALP activity and Mn-MSN-mediated enhancement. Alizarin Red S qualitative detection (Figure 5B) showed that the Gel and Mn-MSN@Gel groups had a small amount of calcium nodule deposition at 14 days. However, after 21 - day culture, calcium nodule formation in the Mn - MSN@Gel group increased significantly compared with the Gel group.

### 3.5 Mn-MSN@Gel promotes the expression of genes related to antioxidant, anti-inflammatory and osteogenic and tenogenic differentiation

**FIGURE 6:**
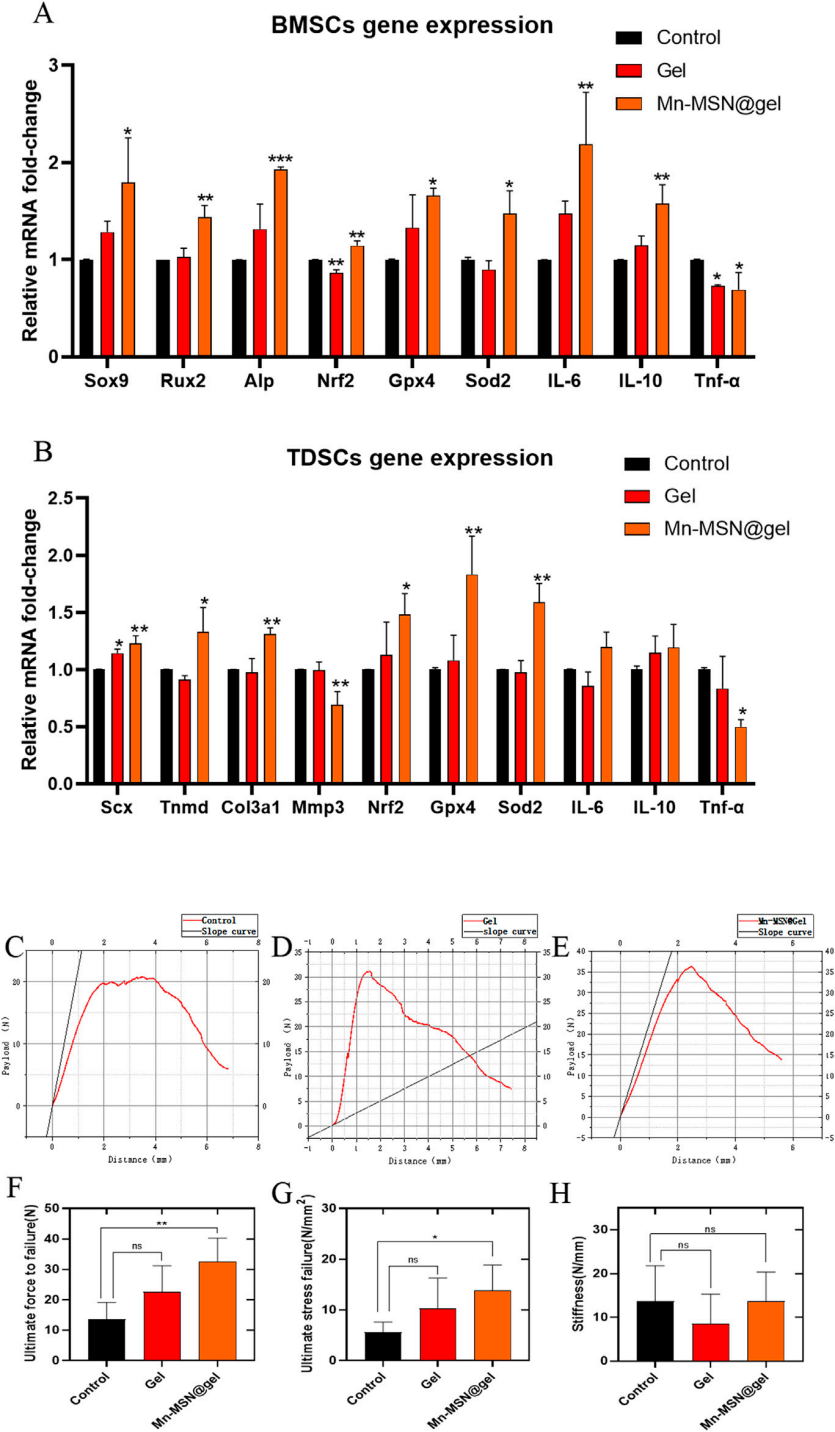
**(A)** Rat-BMSCs Relative gene expression; **(B)** rat-TDSCs Relative gene expression; **(C–E)** The relationship curve (red) between the payload and distance of different groups, and the slope (black) at the starting part represents the elasticity of TBI; **(F)** Ultimate force to failure of different groups; **(G)** Ultimate stress to failure of different groups; **(H)** Stiffness of different groups. (N = 3; *, P < 0.05; **, P < 0.01; ***, P < 0.001).

Figure 6A demonstrates that Mn-MSN@gel significantly enhanced LPS-stimulated rat-BMSCs’ osteogenic differentiation gene expression compared to controls. Relative expression levels of Sox9 (1.8 ± 0.46-fold), Rux2 (1.4 ± 0.12-fold), and Alp (1.9 ± 0.03-fold) showed statistically significant increases. Concurrently, Mn-MSN@gel upregulated antioxidant-related genes Nrf2 (1.1 ± 0.05-fold), Gpx4 (1.7 ± 0.07-fold), and Sod2 (1.5 ± 0.24-fold) with statistical significance. The treatment group exhibited elevated anti-inflammatory cytokine expression (IL-6: 2.2 ± 0.53-fold; IL-10: 1.6 ± 0.19-fold) alongside reduced pro-inflammatory Tnf-α expression (0.69 ± 0.18-fold), confirming Mn-MSN@gel’s dual regulatory capacity in osteogenic differentiation, oxidative stress modulation, and inflammatory response. In LPS-stimulated rat-TDSCs, Mn-MSN@gel promoted tendinogenic differentiation markers Scx (1.2 ± 0.06-fold), Tnmd (1.3 ± 0.21-fold), and Col3a1 (1.3 ± 0.05-fold) while suppressing Mmp3 expression (0.69 ± 0.12-fold), potentially mitigating collagen degradation. Antioxidant genes Nrf2 (1.5 ± 0.18-fold), Gpx4 (1.8 ± 0.34-fold), and Sod2 (1.6 ± 0.16-fold) were significantly upregulated, though anti-inflammatory factors IL-6 and IL-10 remained unchanged. Notably, pro-inflammatory Tnf-α expression decreased substantially (0.5 ± 0.063-fold). These findings collectively demonstrate Mn-MSN@gel’s efficacy in enhancing TDSC differentiation while regulating oxidative and inflammatory pathways.

### 3.6 Biomechanical analysis of rat rotator cuff injury repair model with Mn-MSN@Gel

Biomechanical analysis revealed distinct performance characteristics among experimental groups (Figures 6C–E). The Mn-MSN@Gel group exhibited superior ultimate rupture force (32.5 ± 7.8 N) compared to controls (13.65 ± 5.5 N, P < 0.05), while the Gel group demonstrated intermediate values (22.58 ± 8.6 N) without statistical significance versus controls (P = 0.1256; Figure 6F). Tensile strength measurements followed similar trends: Mn-MSN@Gel achieved 13.8 ± 5.03 N/mm^2^ versus control’s 5.58 ± 2.02 N/mm^2^ (P < 0.05), with Gel group values (10.36 ± 5.91 N/mm^2^) remaining statistically comparable to controls (P = 0.2073; Figure 6G). Elastic modulus analysis showed the Gel group possessed optimal compliance at the bone-tendon interface (8.595 ± 6.71 N/mm), contrasting with Mn-MSN@Gel (13.74 ± 6.57 N/mm) and control groups (13.64 ± 8.13 N/mm), though intergroup differences lacked statistical significance (Figure 6H). These findings confirm Mn-MSN@Gel’s capacity to enhance critical mechanical parameters - ultimate rupture strength and tensile resistance - without compromising native tissue elasticity. The demonstrated mechanical improvements suggest clinical potential for reducing post-repair retear risks and facilitating functional restoration in rotator cuff injuries.

### 3.7 Pathological staining analysis of rat rotator cuff injury repair model with Mn-MSN@Gel

**FIGURE 7:**
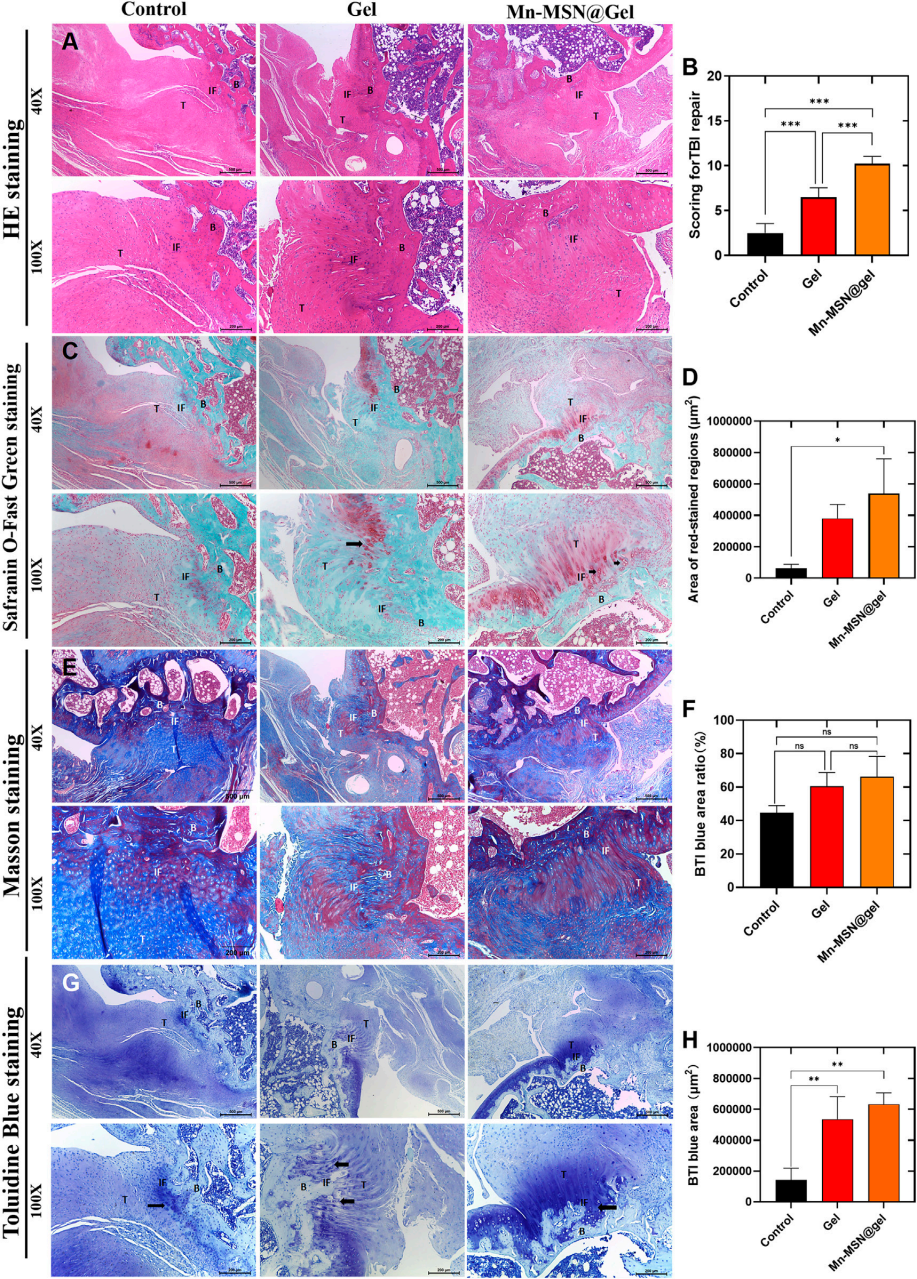
**(A)** H&E stanning of different group; **(B)** Scoring for TBI repair of different group; **(C)** Area of red -stained regions **(D)** Safranin O-Fast Green staining; **(E)** Masson stanning; **(F)** TBI blue area ratio; **(G)** Toluidine Blue staining; **(H)** TBI blue area; (B: Bone, IF: Interface, T: Tendon) (N = 3; *, P < 0.05; **, P < 0.01; ***, P < 0.001).

At 12 weeks post-surgery, H&E staining of rotator cuff injury sites in three experimental groups revealed firm tendon-bone integration with complete gap closure, confirming successful model establishment. The Mn-MSN@Gel group demonstrated superior fiber density, alignment, and maturity at the interface compared to control and Gel groups (Figure 7A). Histological scoring by two blinded evaluators (Z.H. Chen and Z.Y. Du) experienced in tendon pathology showed significant differences: control group 2.5 ± 1.05, Gel group 6.5 ± 1.05, and Mn-MSN@Gel group 10.2 ± 0.84 (P < 0.0001 between all groups, Figure 7B and Table 2). Picrosirius Red/Fast Green staining revealed enhanced fibrocartilage formation in the Mn-MSN@Gel group, exhibiting characteristic tendon-cartilage-bone transitional architecture (Figure 7C, arrow). Quantitative analysis demonstrated significantly larger Picrosirius Red-positive areas in the Mn-MSN@Gel group (538,482 ± 220,849 μm^2^) versus controls (62,906 ± 24,997μm^2^, P < 0.05) and Gel group (379,392 ± 88,963 μm^2^). Masson’s trichrome staining revealed more organized collagen fiber arrangement in the Mn-MSN@Gel group compared to disorganized fibers in controls (Figure 7E). Collagen content analysis showed progressive increases: 44.68% ± 4.3% (control), 60.55% ± 8.1% (Gel), and 66.26% ± 12.1% (Mn-MSN@Gel), though without statistical significance (P = 0.0536 between control and Mn-MSN@Gel groups). Toluidine blue staining confirmed enhanced proteoglycan-rich fibrocartilage formation in treatment groups (Figure 7G). The metachromatic staining pattern, indicative of proteoglycan content (Uehara et al., 2023), revealed significantly larger stained areas in Gel (535,674 ± 145,167 μm^2^) and Mn-MSN@Gel groups (633,045 ± 633,045 μm^2^) versus controls (142,531 ± 75,715 μm^2^, P < 0.01; Figure 7H).

## 4 Discussion

Due to the specificity of combined local drug administration, drug localization and sustained release present significant challenges, underscoring the critical importance of developing biomaterials and compounds for adjuvant therapy. Injectable bio-repair materials with robust tissue adhesion, designed to facilitate gradient transitions between tendons and bone tissues and ultimately achieve mature healing, have garnered increasing attention in scientific research. Hydrogels, hydrophilic polymeric scaffold materials featuring a three-dimensional interconnected porous structure (Ahmed, 2015), exhibit water-swelling behavior without dissolution. Their extracellular matrix-mimetic properties, coupled with prolonged drug retention times and high drug-loading efficiency, render them promising candidates as biomedical drug carriers (El-Sherbiny and Yacoub, 2013). Hyaluronic acid (HA), a naturally occurring non-sulfated linear polysaccharide, has been widely utilized in clinical applications. For osteoarthritis treatment, intra-articular HA injections provide joint lubrication and pain relief (Altman et al., 2015). In trauma repair and tissue engineering, HA promotes cell migration and proliferation, accelerating wound healing (Murakami, et al., 2019). Additionally, HA serves as an effective drug delivery system carrier, enabling sustained drug release via its porous structure and biodegradability (Hemshekhar et al., 2016). However, HA exhibits extremely poor mechanical properties due to its high solubility and rapid *in vivo* degradation. To address these limitations, a dual-crosslinked hyaluronic acid/polyethylene glycol hydrogel (Gel) scaffold was synthesized using HA and polyethylene glycol as raw materials, mal-PEG-mal as a crosslinker, and Diels-Alder click chemistry combined with Schiff base reactions. This approach endowed the Gel with adhesive properties and reduced degradation rates. Further, to enhance mechanical strength and microstructure, mesoporous silica nanoparticles (MSN) and manganese-doped MSN (Mn-MSN) were incorporated into the hydrogel, yielding a manganese-based mesoporous silica nanoparticle-loaded dual-crosslinked hydrogel (Mn-MSN@Gel). Enhancing HA properties while leveraging its inherent advantages is a common strategy. For instance, Liu et al. (Liu et al., 2012) developed a tough, highly stretchable hydrogel by integrating graphene oxide (GO) into the hydrogel network, which resisted fracture even under strains exceeding 3000%. Mesoporous silica (MSN) has attracted extensive research interest owing to its tunable morphology, high specific surface area, large pore volume, thermal stability, and facile surface functionalization (Slowing et al., 2010). Notably, MSN synthesis is cost-effective, scalable, and environmentally abundant. Its porous architecture provides cavities for hosting bioactive molecules or drugs, positioning MSN as a promising platform for small-molecule delivery (Chen et al., 2020). MSN size can be precisely controlled from nanometers to micrometers by adjusting synthesis conditions, enabling broad biomedical applications (Wu et al., 2013). Furthermore, the morphological characteristics of nano-scaffolds significantly influence the adhesion, proliferation, and differentiation of active cells (Mahmoodinia Maymand et al., 2018).

The TBI exhibits slow healing due to challenges in cartilage regeneration and limited vascularization, particularly within fibrocartilaginous regions. Consequently, TBI healing predominantly occurs via type I collagen scar formation (Zumstein et al., 2017; Chen H. et al., 2021; Patel et al., 2018). The inferior biomechanical properties of fibrovascular scars result in immature interfacial healing, significantly elevating risks of reinjury and rupture. Current TBI repair strategies are further limited by deficient gradient structure and disorganized collagen fiber alignment (C et al., 2019). Biomaterial applications frequently focus on regulating osteoinductive capacity through controlled material properties, including chemical composition, surface microporosity, and geometric architecture (Zhang et al., 2014). For instance, pore diameters of 150–500 μm, porosity exceeding 50%, and high pore connectivity have been demonstrated to directly enhance bone mineralization and vascularization (Bai et al., 2010; Mastrogiacomo et al., 2006). Notably, ceramic biomaterials featuring micro/nanostructured surfaces with interconnected macropores significantly improve cell adhesion, proliferation, and *in vitro* osteogenic differentiation of bone marrow mesenchymal stem cells (BMSCs), while augmenting *in vivo* bone regeneration (Lin et al., 2013; Xia et al., 2013). Consistent with these findings, the Mn-MSN@Gel developed in this study demonstrated a suitable three-dimensional mesoporous structure, excellent biocompatibility, and enhanced BMSC adhesion, proliferation, and osteogenic differentiation. The silicon component in Mn-MSN@Gel has been previously reported to exert osteogenic effects (Xia et al., 2016).

Silicon (Si) exhibits immunomodulatory properties during early inflammatory phases post-implantation, suppressing macrophage-mediated pro-inflammatory responses (Huang et al., 2018) and activating monocytes (Song et al., 2020). Our experimental results indicate that Mn-MSN@gel significantly enhances the expression of IL-6 and IL-10 in BMSCs, while reducing Tnf-α expression, thereby exerting an anti-inflammatory effect. As you pointed out, IL-6 possesses dual functions in inflammation—both pro-inflammatory and anti-inflammatory. The co-induction of IL-6 and IL-10 in the BMSC model essentially reflects the dynamic balancing mechanism of the immune system. Milwid et al. reported that IL-6 secreted by BMSCs binds to sIL-6R on surrounding cells, activating the STAT3 pathway, which in turn induces IL-10 secretion and establishes a negative feedback loop to suppress excessive inflammatory responses (Milwid et al., 2012). Furthermore, in a murine arthritis model, elevated serum IL-6 levels indicate the initiation of local inflammation. BMSCs exert a paracrine effect by secreting IL-10 and PGE2, thereby promoting a Th2-type immune shift and systemically steering the immune response toward anti-inflammation (Bouffi et al., 2010). Interestingly, excessive IL-6 secretion has been found to inhibit β-catenin activity, impairing the osteogenic capacity of osteoporotic BMSCs (Li et al., 2016). Neutralizing antibodies against IL-6 have been shown to rescue vertebral osteoporotic phenotypes in mice (Li et al., 2016). Additionally, high levels of IL-6 expression are known to trigger chondrocyte apoptosis, cartilage matrix degradation, cartilage collapse, and synovial infiltration (Noronha et al., 2019). While low levels of IL-6 may induce anti-inflammatory responses, overexpression can exacerbate inflammatory damage. This suggests that IL-6 may play opposing roles in tendon-bone healing, warranting further mechanistic studies to better elucidate its contributions. Additionally, Si stimulates collagen synthesis and organic matrix production through silicate signaling pathway modulation, thereby improving bone mechanical properties and biocompatibility (Reffitt et al., 2003). Si also facilitates tendon-bone healing by enhancing tenocyte proliferation and differentiation, as well as augmenting tendon tissue regeneration (Wan et al., 2024; Gereli, 2014). Corroborating these reports, our experimental results confirmed that Mn-MSN@Gel effectively promoted tendon-derived stem cell (TDSC) differentiation, accompanied by upregulated expression of Col3a1, Scx, and Tnmd. Manganese (Mn), an essential trace element in biological systems, participates in enzymatic catalysis and plays critical roles in redox reactions and antioxidant mechanisms (Burda et al., 2005). Tendon and bone healing are potentially associated with oxidative damage and oxidative stress (Lui et al., 2022; Callaway and Jiang, 2015). Secondly, Mn is involved in various physiological processes, including the regulation of cellular activities (He et al., 2023). For instance, Mn functions directly as a cofactor for several key enzymes involved in metabolic activities such as gluconeogenesis and chondrogenesis (Horning et al., 2015). Moreover, Mn-based materials have demonstrated significant promotive effects on tissue healing (M et al., 2021). Finally, Mn ions possess notable anti-inflammatory properties. For example, Luo et al. found that the degradation and release of Mn ions from metal/metal oxide nanoparticles can alleviate the progression of osteoarthritis through anti-inflammatory effects, cartilage repair promotion, and inhibition of cartilage ossification (Luo et al., 2021). In summary, considering Mn’s antioxidant capacity, its facilitation of tendon-bone healing, and its anti-inflammatory effects, we have selected Mn as the active metallic component in our materials for tendon-bone repair. In this study, the Mn-MSN@Gel demonstrated significant efficacy in promoting cartilage formation in a rat rotator cuff injury model. It alleviated oxidative stress in BMSCs and TDSCs under inflammatory conditions by upregulating antioxidant enzymes (Sod2, Gpx4) and the core antioxidant transcription factor Nrf2, thereby exerting dual antioxidant and cartilage-repair functions. In tendon stem cells (TSCs), downregulation of MMP3 is closely associated with anti-degradation effects, and its regulatory mechanisms involve multi-level signaling cascades and transcriptional regulation. For example, activation of the TGF-β1/Smad2,3 pathway can upregulate anabolic markers in chondrocytes such as Col2a1 and Sox9, while simultaneously downregulating catabolic markers like MMP3 and MMP13 (Rodeo et al., 1999). In addition, inhibition of the Wnt/GSK-3β/NF-κB signaling axis can directly suppress MMP3 promoter activity, thereby ameliorating the progression of osteoarthritis (Ren et al., 2023). Interestingly, inflammatory cytokines also show potential links with MMP3 expression. BMSCs can secrete IL-1ra to block interactions between IL-1α/IL-1β and their receptors, effectively inhibiting the release of MMP3 and chemokines (Ren et al., 2023). In the context of tendinopathy, IL-1β promotes MMP3 expression by activating the MAPK/NF-κB pathway, whereas TGF-β1 exerts anti-degradation effects by suppressing this pathway (Liao et al., 2021). Collectively, the Mn-MSN@Gel that can slowly release Si and Mn elements is a promising multi-mechanism injectable biomaterial for structural TBI healing. Future integration of bioactive factors could further advance its potential as a therapeutic strategy for TBI injuries.

## 5 Conclusion

**FIGURE 8:**
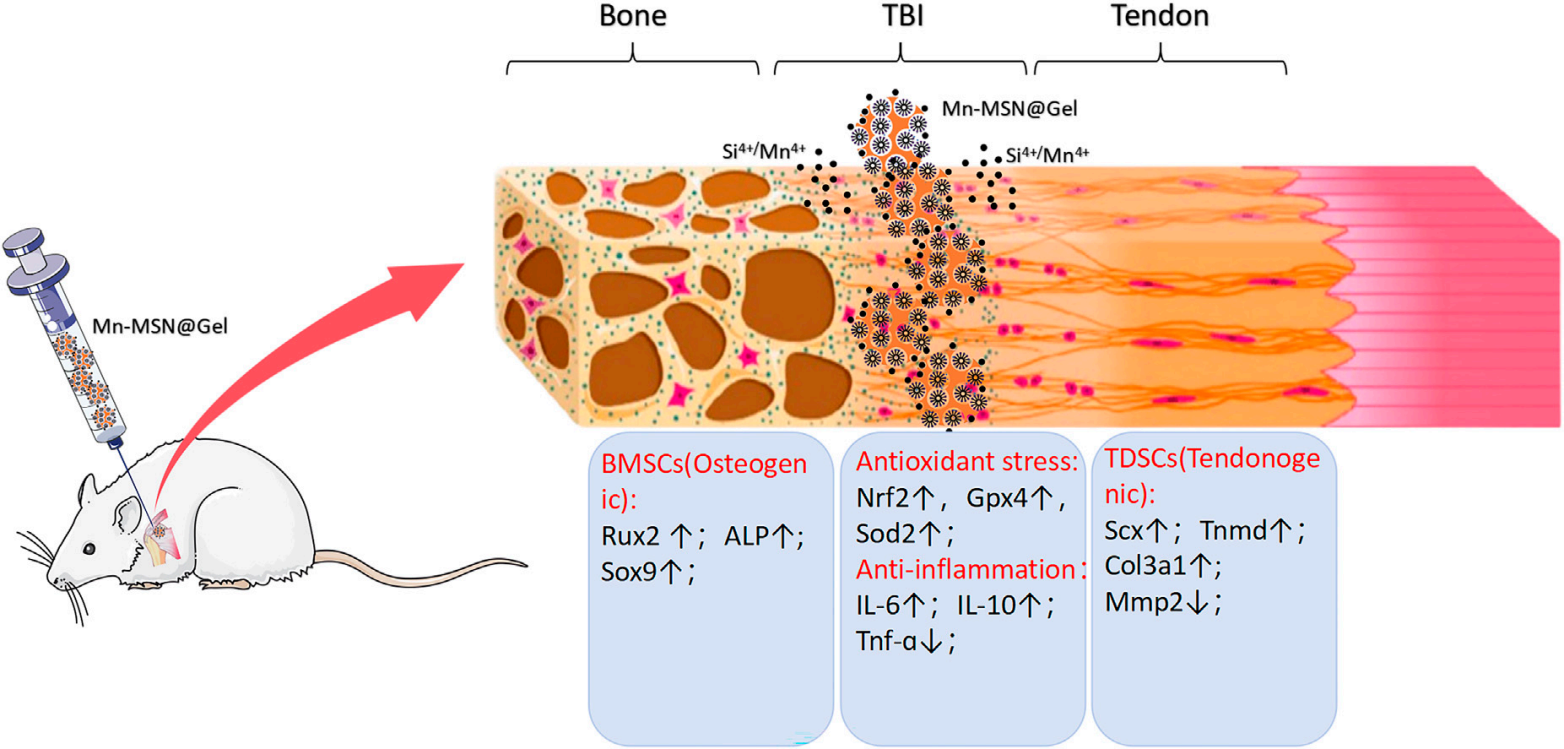
Schematic illustration depicting the porous structure of Mn - MSN@Gel, which facilitates the sustained release of Si^4+^ and Mn^4+^ ions. This promotes osteogenic gene expression in rat - BMSCs and tendon - forming gene expression in rat - TDSCs at the tendon - bone interface (TBI) following rotator cuff injury. Meanwhile, it simultaneously modulates the local inflammatory response and oxidative stress.

The study demonstrated that Mn-MSN@Gel possesses a three-dimensional porous structure with uniform distribution of silicon, oxygen, and manganese elements, enabling sustained slow release of Si^4+^ and Mn^4+^ ions. This hydrogel is facile to fabricate and exhibits favorable mechanical strength, injectability, self-healing capability, degradability, and cytocompatibility. It upregulates osteogenic differentiation genes in rat-BMSCs and tenogenic differentiation genes in rat-TDSCs. Additionally, Mn-MSN@Gel modulates oxidative stress- and inflammatory cytokine-related genes, demonstrating anti-inflammatory effects and mitigating oxidative stress damage (Figure 8). Mn-MSN@Gel injection markedly enhanced biomechanical recovery and stimulated robust tissue regeneration at the tendon-bone interface (TBI) in a rat rotator cuff repair model. These results underscore Mn-MSN@Gel as an innovative biomaterial platform with significant translational promise for achieving structural and functional restoration of TBI in rotator cuff injuries.

## Data Availability

The datasets presented in this study can be found in online repositories. The names of the repository/repositories and accession number(s) can be found below: https://www.ncbi.nlm.nih.gov/, Gene ID: 140586 https://www.ncbi.nlm.nih.gov/, Gene ID: 367218 https://www.ncbi.nlm.nih.gov/, Gene ID: 114108 https://www.ncbi.nlm.nih.gov/, Gene ID: 680712 https://www.ncbi.nlm.nih.gov/, Gene ID: 64104 https://www.ncbi.nlm.nih.gov/, Gene ID: 84032 https://www.ncbi.nlm.nih.gov/, Gene ID: 171045 https://www.ncbi.nlm.nih.gov/, Gene ID: 117519 https://www.ncbi.nlm.nih.gov/, Gene ID: 29328 https://www.ncbi.nlm.nih.gov/, Gene ID: 24787 https://www.ncbi.nlm.nih.gov/, Gene ID: 24498 https://www.ncbi.nlm.nih.gov/, Gene ID: 25325 https://www.ncbi.nlm.nih.gov/, Gene ID: 24835.
